# EZCancerTarget: an open-access drug repurposing and data-collection tool to enhance target validation and optimize international research efforts against highly progressive cancers

**DOI:** 10.1186/s13040-022-00307-9

**Published:** 2022-10-01

**Authors:** David Dora, Timea Dora, Gabor Szegvari, Csongor Gerdán, Zoltan Lohinai

**Affiliations:** 1 Department of Anatomy, Histology, and Embryology, Semmelweis University, Tuzolto st. 58, Budapest, 1094 Hungary; 2 Department of Management and Business Economics, Budapest University of Technology and Economics, Budapest, Hungary; 3 Translational Medicine Institute, Semmelweis University, Budapest, Hungary; 4 National Korányi Institute of Pulmonology, Piheno ut 1., 1121 Budapest, Hungary; 5 Institute of Enzymology, Research Centre for Natural Sciences, Budapest, Hungary

**Keywords:** Drug-repurposing, Cancer research, Drug database, Data mining, Lung cancer

## Abstract

The expanding body of potential therapeutic targets requires easily accessible, structured, and transparent real-time interpretation of molecular data. Open-access genomic, proteomic and drug-repurposing databases transformed the landscape of cancer research, but most of them are difficult and time-consuming for casual users. Furthermore, to conduct systematic searches and data retrieval on multiple targets, researchers need the help of an expert bioinformatician, who is not always readily available for smaller research teams. We invite research teams to join and aim to enhance the cooperative work of more experienced groups to harmonize international efforts to overcome devastating malignancies. Here, we integrate available fundamental data and present a novel, open access, data-aggregating, drug repurposing platform, deriving our searches from the entries of Clue.io. We show how we integrated our previous expertise in small-cell lung cancer (SCLC) to initiate a new platform to overcome highly progressive cancers such as triple-negative breast and pancreatic cancer with data-aggregating approaches. Through the front end, the current content of the platform can be further expanded or replaced and users can create their drug-target list to select the clinically most relevant targets for further functional validation assays or drug trials. EZCancerTarget integrates searches from publicly available databases, such as PubChem, DrugBank, PubMed, and EMA, citing up-to-date and relevant literature of every target. Moreover, information on compounds is complemented with biological background information on eligible targets using entities like UniProt, String, and GeneCards, presenting relevant pathways, molecular- and biological function and subcellular localizations of these molecules. Cancer drug discovery requires a convergence of complex, often disparate fields. We present a simple, transparent, and user-friendly drug repurposing software to facilitate the efforts of research groups in the field of cancer research.

**Supplementary Information:**

The online version contains supplementary material available at 10.1186/s13040-022-00307-9.

## Introduction

There is an increasing need for open-access drug repurposing databases for researchers in the translational and clinical field due to the emergence of many potential new therapeutic targets every year. This trend will likely continue in the future with exponentially increasing repositories full of data, and data mining requires particular expertise and qualifications that cannot be expected from a single group of researchers. The development of novel pharmaceuticals takes enormous effort and immense financial and human resources, a decade of research and clinical trials until approval with more advancements in prevalent cancers. Research groups usually select their target and related research directions without cross-optimization of efforts across individual groups. Industry AI-based drug developments have limitations and also require preclinical validation. Therefore, in silico approaches with solid human resources might assist than fully overtake all steps.

The therapeutic potential of a molecular target, the suitable inhibitor (or agonist), might already be available in another indication, or a small molecule lead compound is available that needs further modification and preclinical testing. Nowadays, a wide selection of databases is available for various goals in drug repurposing [1–3]. Omics data about molecular targets can be retrieved from *Uniprot* [4]*, Genecards* [5]*, TTD* [6]*, STITCH* [7]*, BioGRID* [8]*, and STRING* [9]*,* or corresponding pathways from *KEGG* [10]*, Pathways Common* [11], or *Reactome* [12] with to a certain level of deficient or overlapping information*.* Information about drugs launched or in development (drug omics data) can be obtained from *Pubchem* [13]*, Drug Bank* [14]*, Drug Map Central* [15], or *FDA or EMA Label* repositories. Also, the Clinicaltrials.gov, SIDER [16], or FDA Adverse Event Reporting System (FAERS) platforms provide essential information on stages of drug testing.

Platforms for browsing and visualizing drug-target interactions and drugs in disease-context are already available for many users, such as *Drug Target Profiler* [17], *Cancer Genome Interpreter* [18]*, SwissTargetPrediction* [19]*, OpenTargets* [20], and *PharmGKB* [21]*.* These platforms include many entries and a gargantuan amount of unstructured information that is sometimes time-consuming and difficult to handle, especially for those lacking expertise in the field. The data mining techniques require specific processes usually nonexistent in less prevalent cancers and often lack financial incentive for clinical testing [22]. Cancer drug discovery requires a convergence of complex, often disparate fields. There is a great need for simple, transparent and user-friendly drug repurposing databases.

One recent endeavor with high impact is Broad Institute's Clue.io, which serves as a drug repurposing hub curated and annotated collection of FDA-approved drugs, clinical trial drugs, and preclinical tool compounds with a companion information resource [23]. In this paper, we present a novel, open access, data-mining, drug repurposing platform, deriving our searches from the entries of *Clue.io. EZCancerTarget* provides researchers and clinicians (especially in cancer research) an easy-to-use platform to retrieve all the necessary information for a freely selected array of potential therapeutic targets with a parallely-working, data-mining application. In addition, *EZCancerTarget* provides detailed biological information on the selected set of target molecules using open-access databases, such as Uniprot, GeneCards, Gene Ontology and STRING. This way, the user receives a concise summary on the biological relevance of every target, that is explicitly important for researchers who are not experts in molecular biology.

## Methods

### Clue.io and dataPatch R script

Target inclusion/exclusion depends on search results from a clue.io query. *EZCancerTarget* consists of 3 separate R scripts. The first script -clue.R- calls various clue.io REST API endpoints to build up a result table**.** If the main API call does not find any component for a target, that target will not be involved in further processing steps since no known drug repurposing approaches are available in Clue.io. R looks up the input target list in two ways. First, it tries to access a private and/or shared Google Sheet file. It requires a unique "key" (a token) given to clue.R via a simple environmental variable. If this secret key is available for the script, it authenticates by *gargle* package [24] to access Google Sheet API services. Next, it reads the sheet and takes the values from its first three columns. An ID string also identifies the Google Sheet, and it is passed via an operating system environment. If there is no API key/Google Sheet identifier, then clue.R tries to load a TSV file from the INPUT directory of the EasyCancerTarget directory. Clue.R merges the outputs of various clue.io API calls and saves the composing table into an RDS file (R-specific data format to store and load R objects). At the next stage, data patch.R reads this RDS file and restores the data frame from it.

### PubMed function

This function searches for the compound name received from clue.io and sends a search request to PubMed® service of *National Center for Biotechnology Information* (NCBI). It restricts the result set by including only clinical trials, meta-analyses, randomized controlled trials, reviews and systematic reviews. The results are ordered by the best match algorithm of PubMed®. Our function picks the top 3 of the result set and stores it in the global datatable. If there is no hit at all, dataPatch.R provides the used search URL and this search can be re-initiated and/or refined by users of *EZCancerTarget*. pubMed function uses a simple XPath query to extract PubMed identifiers embedded into the resulting HTML source code.

### XmlUniProt function

This function collects data from the *UniProt* website. *UniProt* provides APIs to access and query its data. Easy to access the human readable contents in machine readable formats (for example XML, RDF, etc.). The usage of the *UniProt website REST API* is straightforward, since the input target list also contains UniProt identifiers. xmlUniProt extracts GO (*Gene Ontology*) molecular function and cellular component terms, *STRING* and *Reactome* references from the received XML data. These specific entries are stored in simple R lists and added to the already collected data in a new column: “UniProtData”.

### Presentation layer

A web browser is a "mandatory" software of each end user's computer, so HTML is a clear choice to summarize, visualize and deliver collections of texts, images and hyperlinks. An important part of this rendering is building an HTML source file and populating it with the collected data in an user-friendly way. *EZCancerTarget* follows the popular *Model-View-Controller* design pattern even though it composes only static HTML output (View) from the data (Model) at this stage of the workflow. *(NOTE: However previous actions and functionalities of the workflow can be interpreted as the Controller part of the MVC pattern).*

Further detail on methods and functions is available at https://cycle20.github.io/EZCancerTarget/methods.html. All the R scipts and versions' descriptions and runtime environment are freely accessible at: https://github.com/cycle20/EZCancerTarget.

## Results

### Construction and content

**Fig. 1:**
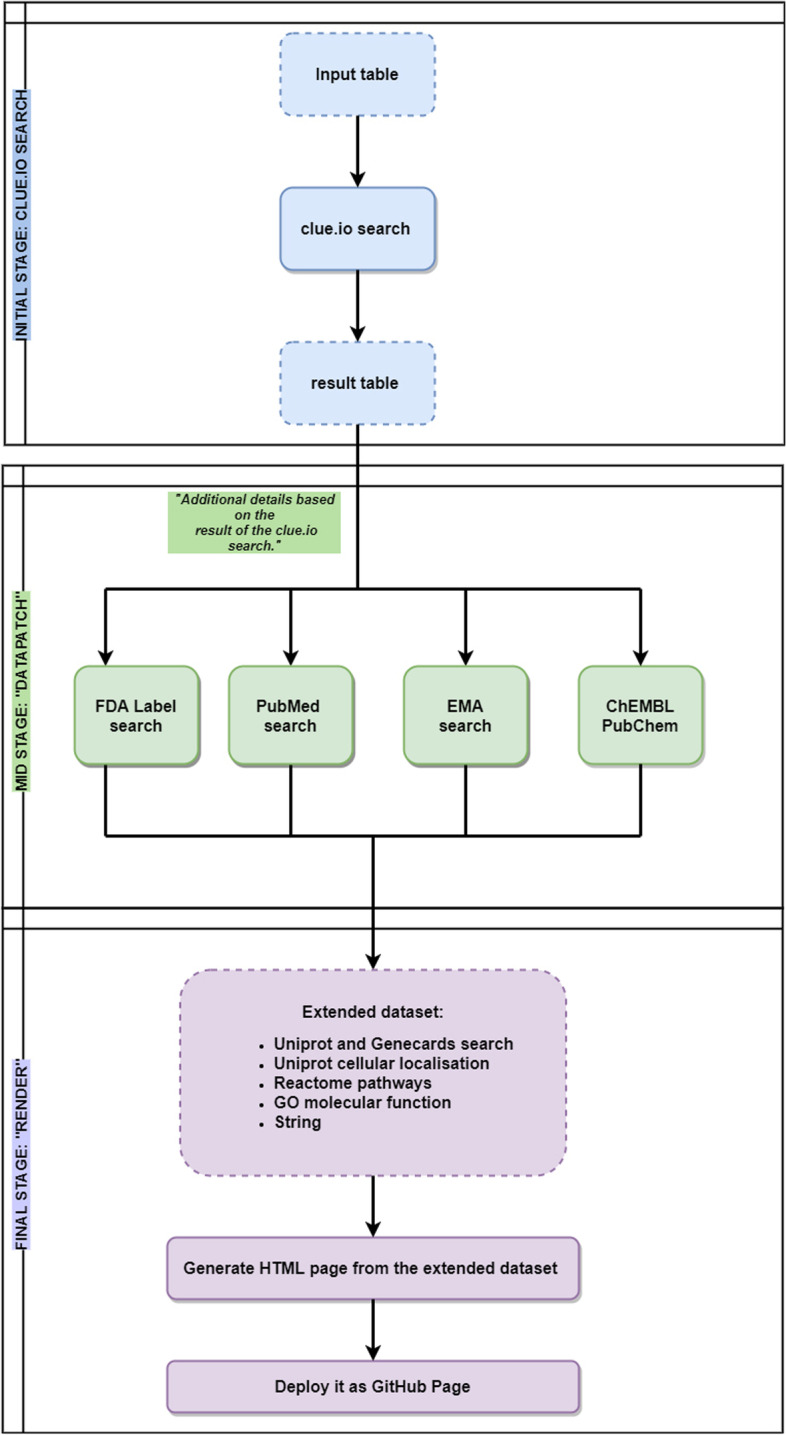
Flowchart of functionality. Flowchart describes the main steps of *EZCancerTarget*’s functionality, including data input, Clue.io target search, cross-referencing in databases (Datapatch) and molecular background information on selected targets (Render)

Each entry obtained from the search results in the interactive online platform of *EZCancerTarget* is referenced and has at least one scientific piece of evidence. Figure 1 shows a flowchart on the processing steps and workflow of *EZCancerTarget*. First, users of *EZCancerTarget* can start their workflow by opening the project's starting page on Github (https://cycle20.github.io/EZCancerTarget/). Then, users can upload their target list with three pieces of information into a google spreadsheet directly (Target INPUT), which requires a quick authorization step from the project administrator (to avoid interference from simultaneous and multiple users), or can manually install it in R environment. Installation process is described in detail on the aforementioned Github page.

EZCancerTarget fetches its input from a simple data source. It can be a TSV file in case of running scripts on user-controlled computer. Another option is updating a shared Google Spreadsheet that is processed by workflow scripts on *GitHub*. EZCancerTarget load data from *clue.io*, then download data from other sources: *FDA Label* service, *PubMed*, *EMA*, *ChEMBL*, *PubChem* (Fig. 1). After data collection it generates a user-friendly report file and a summary output data file. The report HTML file can be opened by a browser. If the workflow runs on *GitHub*, it will deploy the report file as a public GitHub web page.

**Fig. 2:**
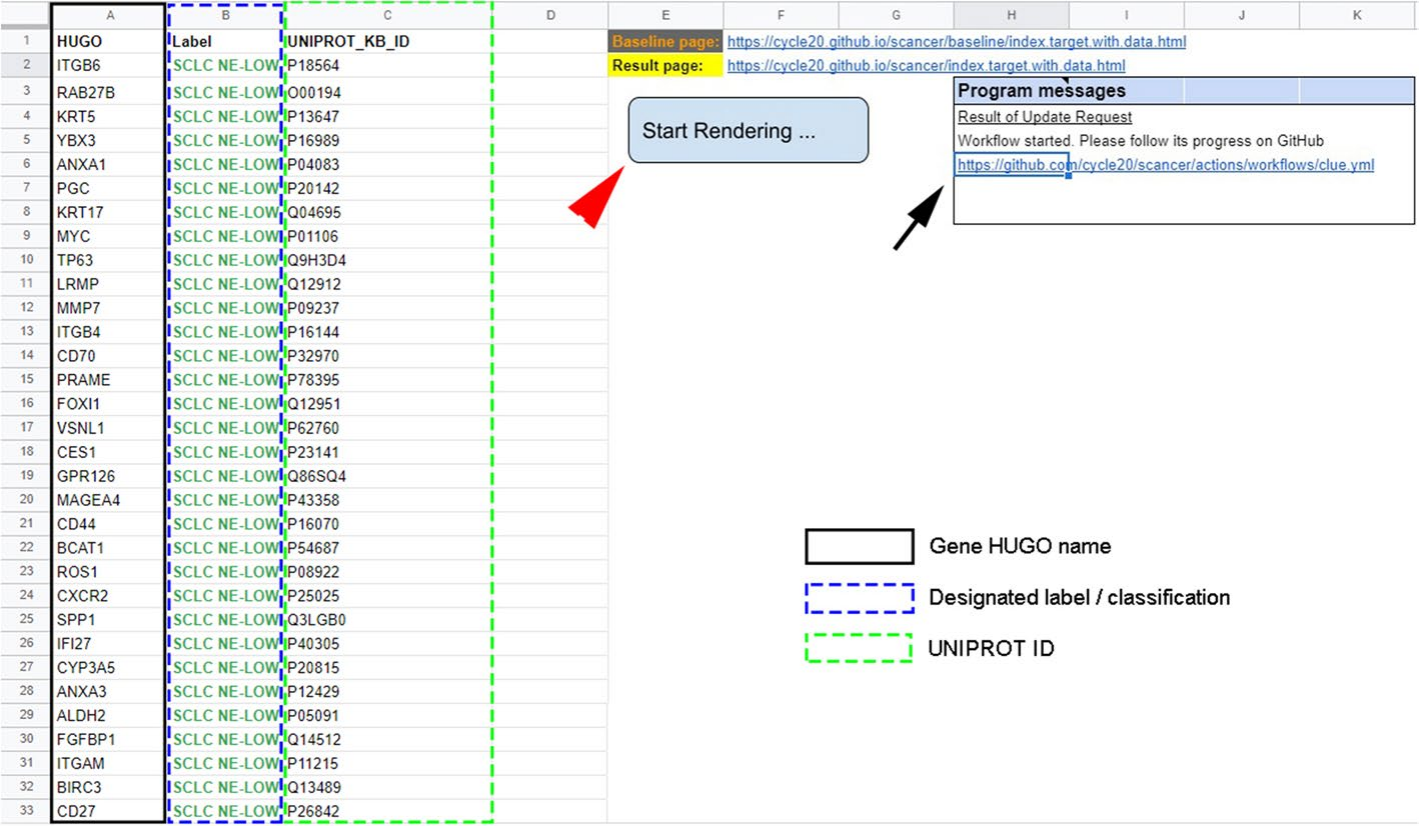
Input table for molecular targets. Users can enter selected targets’ HUGO name (black rectangle), label (blue dashed rectangle) and Uniprot ID (green dashed rectangle) in columns **A**, **B** and **C**. Hitting “Start Rendering” will initiate the Clue.io search (red arrowhead). Progress can be traced by clicking on hyperlink in cells H6-K6 (black arrow). Clicking on the hyperlink in cell F2-I2 reveals the results page

In the input table, the first piece (Fig. 2 Column A), asks for the HUGO ID. In column B, users can give a "Label" for every target for classification and clustering, useful in later work. The third piece (Fig. 2 column C) is the UniprotKB ID of the searched gene. Inputs for the HUGO and Label columns are limited to 12 characters. On the right side of the spreadsheet (columns E-K), hyperlinks provide access to the results page on Github, and in the "Results of Update Request" box, users can check the query's status. Hitting the "Start Rendering" button located on columns E–F starts the query. The area within E1-K8 are protected and automatically overwritten if edited (Fig. 2).

By clicking on the "Result page" link on the target spreadsheet, we can access the results of our query within approximately 30 min. Clicking on the hyperlink in cell H6 we can follow the progress of the query (Fig. 2, arrow). A new query overwrites the earlier one in the web application, but every previous version is saved on Github under the "Result of Update Request" link (https://github.com/cycle20/EZCancerTarget/actions/workflows/clue.yml). A scrollable panel displays all the targets on the left with at least one valid drug compound available. The software automatically excludes entries where no drug or small molecule inhibitor/agonist is available according to the Clue.io repurposing hub.

In the first entry of the results list ("summary") the evaluation report on the search is accessible. In the "overview" section, it displays the total number of found compounds for all listed targets and the average number of compounds per target. The amount of found compounds are classified according to their pre- and clinical phase as well. In the "molecular background" section details from the retrieved molecular background data is evaluated according to the number of found Reactome- or KEGG pathways and subcellular localizations, String interactors and GO molecular functions, biological processes and. Finally, every listed target is separately detailed of their compound entries in PubMed, PubChem, ChEMBL and DrugBank.

**Fig. 3:**
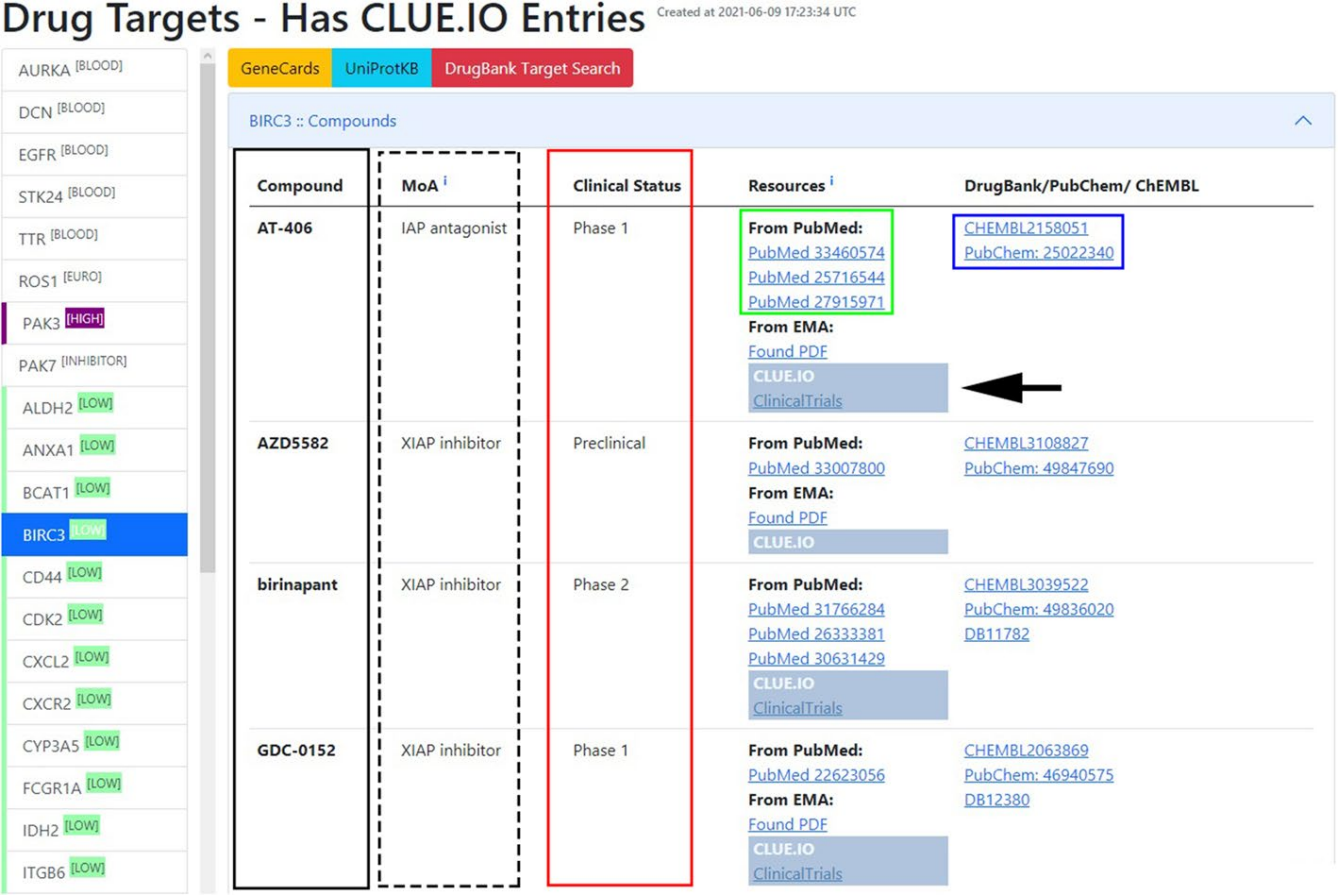
List of drug targets. Clicking on the labels of selected targets (column on left side) unveils available compound list (black box) describing also mechanism of action (MoA, dashed box), clinical status (red box), resources of information on PubMed (green box) and DrugBank/PubChem/ChEMBL entries (blue box)

The platform creates a table for every target, where different columns indicate the mechanism of action (MoA), clinical status (preclinical, phase 1, phase 2, phase 3, or launched), and the search resources from PubMed EMA and the direct entry from Clue.io. Furthermore, the query table includes hyperlinks with DrugBank, PubChem, and ChEMBL IDs to quickly access the compounds' chemical and pharmacological properties (Fig. 3).

**Fig. 4:**
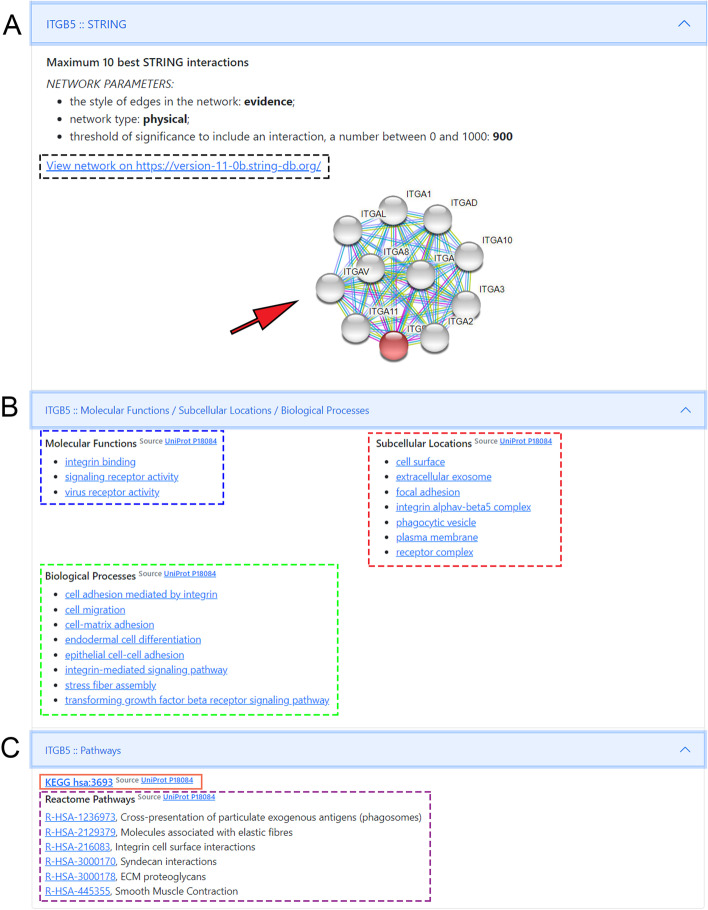
Details on the molecular background of druggable targets. **A** shows the network map from String.db with static string map and hyperlink to String-db entry. **B** displays hyperlinks to “molecular function”, “biological processes” and “subcellular localisation” to browse the UniProt database on molecular background. By clicking directly on the titles, we can access a specific function. **C** shows hyperlinks to visualize “KEGG” and “Reactome” pathways of the selected target. For Reactome, clicking on individual pathway titles we can directly access the infographic of the given pathway

*EZCancerTarget* also gives a comprehensive, highly structured overview of the selected targets regarding their molecular biology data, including molecular function (Gene Ontology), their connectome (STRING), participation in pathways, and cellular localization (Reactome and KEGG) retrieved from various databases. Hyperlinks to GeneCards and DrugBank Target Search are also available but differently structured as for UniProt entries. The "STRING" entry opens a static string map for the target and provides a hyperlink to string-db.org (Fig. 4). The following entry carries the "Molecular Functions / Subcellular Localisations" title, where the two main hyperlinks (source) lead to UniProt's "Function" and "Subcellular Localization" pages. Molecular function entries and target localizations are also provided as text separately, where hyperlinks lead to the QuickGo platform to obtain further information about relevant compartment-specific molecular pathways (Fig. 4). The last entry named "Pathways” provides links to every Reactome database, where the target's participation is visualized in every relevant metabolic pathway (Fig. 4). The hyperlink to the entry of the KEGG database is also displayed here, without being broken down to individual links to pathways. Supplementary Video 1 shows a short tutorial about the functionality of the program and the main steps to generate a query.

### Current content

This current content includes 145 molecular that focus on the three most aggressive malignities with limited therapeutical prospect: small-cell lung cancer (SCLC) [25], triple-negative breast cancer (TNBC) [26–28] and pancreatic adenocarcinoma (PADC) [29–31]. The starting content provides a template for researchers and serves as an example to demonstrate the software's functionality. The list can be further expanded or replaced with any UniProt-listed gene and can be customized to the needs of the research group using the platform. Users can also give titles to genes in the "label" column to address targets and classify them into different groups. When a researcher would like to use the platform, they can edit the gene list according to their goals after giving the administrator access to the spreadsheet. The original content is saved in the github main page (https://cycle20.github.io/EZCancerTarget), therefore can be retrieved and reset anytime. When the researcher finishes constructing their target list, the administrator refreshes the cache for the optimization of the search and generates a new result page. The results page displays a "summary" section, where the list of target genes with- or without relevant drug repurposing entries (retrieved from clue.io) are available. Molecular background data is only displayed for genes with at least one valid clue.io compound entry.

## Discussion

Open access journals and databases are an essential basis for drug target developments. Endeavours, like the TCGA database and Oncomine [32] has contributed vastly to accelerate drug research in oncology the latest decade and concurrently multiple other enterprises emerged to assist researchers with valuable genomic, transcriptomic and proteomic data in the pursue for novel cancer biomarkers [33–36]. However, the information is not well structured for specific diseases, including rare and highly progressive cancers [37, 38]. Moreover, it is challenging and time-consuming to associate the latest biomarkers with drugs to pick the optimal way and contribute to the field. Only a few research groups with diverse expertise can participate, leaving many researchers without an equal opportunity of involvement. Also, the individual interest of these groups might not represent the optimal way to examine diseases. Therefore, we propose a novel, optimal target selection methodology. Notably, after the success of PD-L1-inhibitors in non-small cell lung cancer (NSCLC), where 5-year-survival in extensive-stage disease increased from 2 to 25%, there has been a keen interest to expand on immunotherapy utilization in small cell lung cancer (SCLC) as well. Two anti-PD-1 immunotherapies, nivolumab and pembrolizumab, have had their FDA approval [39, 40], but they were withdrawn after the confirmatory phase III trials, because statistical significance for overall survival was not significant compared to control groups. Nevertheless, PD-L1 expression in SCLC has never been unequivocally correlated with the response.

Open-access data on the latest research requiring further validation is of high interest to the field. In low-prevalence and highly aggressive cancers, scarcity of available tissue samples limit research, so there is an unmet need to share and optimize resources in the field. It has been decades with only modest therapeutic advancements for highly progressive cancers such as pancreatic cancer, triple-negative breast cancer, glioblastoma multiforme, or SCLC, with an unmet need for advances. To enhance drug target development, we believe that *EZCancerTarget* can serve as an easy-to-use, semi-comprehensive data-mining platform for drug-repurposing and can assist significantly smaller research groups in the fight against malignancies. EZCancerTarget only provides a tool for researchers to keep their target list organized and to aid these researchers in selecting the best targets to start validation with. Our software aims to assist the decision-making process of these projects druggable and clinically relevant targets for validation.

For example, our previous study revealed a set of molecular targets showing overexpression in immun-infiltrated SCLC [25], which might be clinically valuable for targeted immunotherapies in the future. However, not every target molecule is readily druggable, or clinical trials with small molecule inhibitors might have already failed against a selected substance. This was the case with MMP7, a matrix-metalloprotease exhibiting upregulation by manifolds in infiltrated SCLC. EZCancerTarget readily provided information with PubMed links to the failed Phase III clinical trial (performed almost 20 years ago), proving that its inhibitor, marimastat, is ineffective in this type of cancer. In contrast, in the case of molecular target ITGB6 (Integrin-beta 6) from the same screen [25], the software presented us with an available integrin antagonist compound, GSK3008348, used for an utterly different indication (pulmonary fibrosis), but with successful results. Through the STRING interaction, subcellular localization and pathway entries provided by EZCancerTarget, we learned that ITGB6 is structurally and functionally connected to the tumor microenvironment's proteoglycan components. Tenascin (TNC) was shown to be one of its closest interactors, whose role in lung cancer as an immunosuppressive agent promoting tumor recurrence has been reported by multiple studies [41, 42]. Since there is no available substance under study for the direct inhibition of TNC (source: clue.io), targeting ITGB6 might impede the whole pathway, promoting cancer propagation, making ITGB6 a druggable and biologically valid target.

Protein expression, subcellular localization and involved biological pathways matter for comprehensive experimental validation and clinical context. Altogether, if a research team finds a molecular target for validation that has already clinically proven pharmaceutical agonists/antagonists, it is still essential to put the molecule in a biological context that Clue.io does not provide alone. EZCancerTarget has a filter function and does not display targets with at least one available compound supported by a preclinical study or a clinical trial. Thus, researchers begin with a target list already narrowed down based on drug availability.

The landscape of cancer research has been transformed by open-access genomic, proteomic databases, such as The Cancer Genome Database (TCGA) [43], Oncomine [32], Human Protein Atlas [44], Cancer Cell Line Encyclopedia [45] or DepMap [46] and a multitude of drug-repurposing databases [3]. Still, casual users can face difficulties orchestrating high-throughput screenings and interpreting the vast amount of data generated using these platforms. Furthermore, researchers require the assistance of an expert bioinformatician to conduct systematic searches and data retrieval if multiple targets are eligible, which is not always feasible for smaller research teams.

Open-acces EZCancerTarget obtains information based on the search engine of the groundbreaking drug-repurposing platform Clue.io. However, on the level of a casual user (like most researchers in the biomedical field without coding or IT experience), Clue.io can provide crisp information on one target at once and the users need to go through one-by-one their target list. In contrast, our software can scan through hundreds of targets simultaneously and provide the same information as Clue.io, but supplemented with references citing the relevant literature of preclinical studies or clinical trials. Sources regarding the biochemical details of the found compounds with their DrugBank, PubChem, and ChEMBL entries are also directly available for the users with one click. Another valuable addition to our software is that it provides a comprehensive overview of the molecular background of every target with an eligible search result. A concise and organized knowledge bank of these targets' pathways, molecular- and biological functions, and subcellular localization can serve educational purposes, but can also enhance the decision-making process for planned preclinical- or clinical validation studies.

An outstanding advantage of *EZCancerTarget* compared to other drug-repurposing platforms is that it does not require any experience in database handling and provides information on the biological background of our target molecules in a processed way that is easy to understand. The latter feature can be used for educational purposes as well. We believe that *EZCancerTarget* is a useful addition to the field of drug-repurposing in cancer science and oncology and will be particularly useful for smaller research groups with limited expertise in database handling.

## Supplementary Information

**Additional file 1.**

## Data Availability

All the R scipts and description of versions and runtime environment is freely accessible at: https://github.com/cycle20/EZCancerTarget.

## Abbreviations

SCLC: Small-cell lung cancer
EMA: European Medicines Agency
TTD: Therapeutic Target Database
STITCH: Sequencing To Imputation Through Constructing Haplotypes
KEGG: Kyoto Encyclopedia of Genes and Genomes
FDA: U.S Food and Drug Administration
SIDER: Side Effect Resource
FAERS: FDA Adverse Event Reporting System
API: Application Programming Interface
REST: Representational State Transfer
MoA: Mechanism of Action
NSCLC: Non small-cell lung cancer
TNBC: Triple Negative Breast Cancer
PADC: Pancreatic Adenocarcinoma
PD-L1: Programmed Death Ligand 1
